# Molecular Mechanism During Mycelium Subculture Degeneration of *Volvariella volvacea*

**DOI:** 10.3390/jof11010007

**Published:** 2024-12-25

**Authors:** Lidan Feng, Lujuan Wang, Yuanxi Lei, Jie Li, Fengyun Zhao

**Affiliations:** 1 College of Food Science and Engineering, Gansu Agricultural University, Lanzhou 730070, China; fengld@gsau.edu.cn (L.F.); 18730059795@163.com (L.W.); 18394309957@163.com (Y.L.); 2 College of Forestry, Gansu Agricultural University, Lanzhou 730070, China; lj81658@gsau.edu.cn

**Keywords:** *Volvariella volvacea*, strain degeneration, transcriptomics, substrate degradation, amino acid metabolism, reactive oxygen accumulation

## Abstract

Periodic mycelial subculture is a method commonly used for the storage of edible mushrooms, but excessive subculturing can lead to the degeneration of strains. In this study, the *Volvariella volvacea* strain V971(M0) was successively subcultured on PDA medium every 4 days, and one generation of strains was preserved every 4 months. Thus, five generations of subcultured strains (M1–M5) were obtained after 20 months of mycelial subculturing, their production traits were determined, and transcriptomic analysis was performed using RNA-seq; the differentially expressed genes were verified via RT-qPCR. The results showed that as the number of subcultures increased, the diameter of the mycelium and biological efficiency gradually decreased; in addition, the time in which the primordium formed increased and the production cycle was lengthened, while strains M4 and M5 lacked the ability to produce fruiting bodies. There were 245 differentially expressed genes between the M1–M5 and M0 strains, while the highest number of differentially expressed genes was between M3 and M0, at 1439; the smallest number of differentially expressed genes was between M2 and M0, at 959. GO enrichment analysis showed that the differentially expressed genes were mainly enriched in metabolic processes, organelle components, and catalytic activities. KEGG enrichment analysis showed that the differentially expressed genes were mainly enriched in metabolic pathways. The further annotation of differentially expressed genes showed that 39, 24, and 24 differentially expressed genes were related to substrate degradation, amino acid synthesis and metabolism, and reactive oxygen species metabolism, respectively. The downregulation of the related differentially expressed genes would lead to the excessive accumulation of reactive oxygen species, inhibit nutrient absorption and energy acquisition, and lead to the degradation of *V. volvacea*. These findings could form a theoretical basis for the degeneration mechanism of *V. volvacea,* and also provide a basis for the molecular function study of the genes related to strain degradation.

## 1. Introduction

Edible mushrooms, also known as macro fungi, are nutrient-rich and environmentally friendly crops that possess a visible fruiting body, which can grow above or below the ground [1]. Since ancient times, humans have used edible mushrooms as food and medicine [2]. *Volvariella volvacea* is one of the most widely cultivated edible mushrooms in tropical and subtropical regions [3]. The annual yield of *V. volvacea* in China is approximately 330,000 tons, accounting for more than 80% of the world’s yield [4]. *V. volvacea* tastes delicious and is rich in protein, fat, amino acids, and other nutrients, and contains medicinal ingredients such as anti-tumor polysaccharides, immune-suppressing proteins, and immunomodulatory lectins, which can lower blood pressure and cholesterol [5].

The quality of edible mushroom strains determines the production of the fruiting body and is one of the key factors that affect the economic value of the product [6]. Subculturing is a strategy commonly used for the propagation and preservation of strains, but repeatedly performing subculturing can affect the growth of edible mushrooms and even lead to the mutation or degradation of strains [7], and *V. volvacea* is no exception. *V. volvacea* is a high-temperature edible mushroom, so the storage of this mushroom at 15 °C caused low-temperature damage, while storage at 4 °C for more than 8 h leads to death via autolysis [8]. Therefore, *V. volvacea* cannot be stored at low temperatures like other edible mushrooms, which means that the subculturing is performed more frequently in this stain, resulting in its serious deterioration.

The frequent degradation of strains has long been a serious problem in the production of edible mushrooms, but little is known about the specific causes of strain degradation. However, with the widespread application of high-throughput sequencing technology, the regulation mechanism involved in the rapid growth of *V. volvacea* was revealed via transcriptomics in 2020 [9]. By using the full-length genome data for *V. volvacea*, which were completed in 2013 [8], it became possible to determine the mechanism via which strain degradation occurs during the subculturing of *V. volvacea*. In this study, we obtained subcultured strains of *V. volvacea* with different degrees of degradation via continuous subculturing. Then, the production traits of these stains were measured and the differentially expressed genes were analyzed via RNA-seq sequencing in order to explore the molecular mechanism of degradation, to provide insights into controlling and delaying the degradation of *V. volvacea* strains.

## 2. Materials and Methods

### 2.1. Strains

The commercial *V. volvacea* strain V971(M0) was purchased from the Edible Mushroom Research Institute of Jiangsu, China. In this experiment, a 1 cm^2^ tip mycelia agar piece was inoculated on a potato dextrose agar (PDA) plate every 4 days; this was then cultivated for 20 months using the same methods. Every 4 months (120 days), the strains were obtained after the last subculture and stored on the inclined surface of PDA test tubes. Five subculture strains were obtained using this method, and denoted as M1–M5 [10] (Supplementary Figure S1). All the strains were injected into sterile liquid paraffin and stored at a constant temperature of 18 °C.

### 2.2. Medium Preparation

PDA medium contains 200 g of fresh potato, 20 g of agar, 1.0 g of KH_2_PO_4_, 1.0 g of MgSO_4_·7H_2_O, and 1000 mL of distilled water.

Seed medium contains 88 g of cottonseed hulls, 10 g of bran, 1 g of gypsum, 1 g of lime, and 1000 mL of water. The pH of the medium is 8–9.

Culture medium contains 97 g of waste cotton, 3 g of lime, and 1000 mL of water. The pH of the medium is 8–9.

### 2.3. Mycelium Phenotype

The M0–M5 strains were inoculated on PDA medium plates and incubated at 30 °C for 3 d. The mycelium phenotype was photographed and recorded.

### 2.4. Cultivation of V. volvacea

M0-M5 strains were inoculated in culture frames (30 cm × 22 cm × 10 cm) with 1.2 kg sterilized cultivation substrate. The cultivation and management strategies were previously outlined by Hou, Li [5], with the mushrooms being harvested when the fruiting body grew to the egg-shaped stage. The production cycle and biological efficiency were recorded.

The time required between inoculation and the primordium formation was recorded as the time of primordium formation (d). The time between inoculation and development into the egg-shaped stage was recorded as the production cycle (d). The biological efficiency was calculated according to Equation (1).

(1) $$\mathrm{Biological efficiency}(\%)=\frac{\mathrm{Fresh weight of fruiting bodies}}{\mathrm{Dry weight of cultivation substrate}}\times 100\;\%$$

### 2.5. Sequencing Analysis of Transcriptomics

#### 2.5.1. RNA Extraction and Sequencing

After activation, the M0–M5 strains were inoculated on PDA medium plates and cultured at 30 °C for 5 days. The mycelium on the surface of the medium was gently scraped with a sterile scalpel and then ground into a powder in liquid nitrogen. The total RNA was extracted using the Trizol method according to the instructions of the kits. The purity of the RNA and its quantity were evaluated using a NanoDrop 2000 spectrophotometer((Thermo Scientific, Waltham, MA, USA)). The integrity of the RNA was assessed using an Agilent 2100 Bioanalyzer, and the qualified RNA was sent to Shanghai Ouyi Biotechnology Co., Ltd. (Shanghai, China). Three samples for each strain, amounting to a total of 18 samples, were sent. High-throughput sequencing was performed using the Illumina HiSeq 2500 sequencer (San Diego, CA, USA). The raw data were filtered to remove the joint sequences and low-quality reads, and high-quality clean reads were obtained.

#### 2.5.2. Functional Annotation and Enrichment Analysis of Differentially Expressed Genes

The *V. volvacea* V23 genome (https://mycocosm.jgi.doe.gov/Volvo1/Volvo1.home.html (accessed on 22 December 2024)) was used as a reference, with HISAT2 software (V2.2.1) being employed to compare the reference gene and the clean read annotations [11]. The gene expression of each strain was analyzed using DESeq software (21.10.1). Differentially expressed genes were screened using *p* < 0.05 and |log2 Fold Changes|> 1 as the threshold, and the differentially expressed genes that were obtained and analyzed using GO and KEGG enrichment [12,13].

#### 2.5.3. Real-Time Quantitative PCR Validation

Twelve differentially expressed genes were selected for the RT-qPCR analysis, and the glyceraldehyde-3-phosphatedehy drogenase (GPD) gene was used as the internal reference gene. Primer 7.0 software was used to design the primers. The primer sequences are shown in Table 1. Once the uniqueness had been confirmed, the primers were synthesized by Tsingke Biotechnology Co., Ltd. (Qingdao, China). The reaction was carried out on a LightCycler^®^ 480II fluorescence quantitative PCR instrument (Roche, Swiss, Basel, Switzerland) using the Cham Q SYBR qPCR Master Mix kit (Acres Biotechnology Co., Huaihua, China) [14]. The relative gene expression was calculated using the 2^−ΔΔCt^ method [15].

### 2.6. Data Processing

The indicators of each strain were determined in 3 groups, and the results were expressed as mean ± standard deviation (SD). Microsoft Office Excel 2013 and Tbtools 2021 were used for data statistics and graphing, and SPSS 24.0 was used for one-way ANOVA; *p* ≤ 0.05 indicated a significant difference.

## 3. Results and Analysis

### 3.1. The Change in Mycelial Character of V. volvacea Subculture Strains

**Figure 1:**
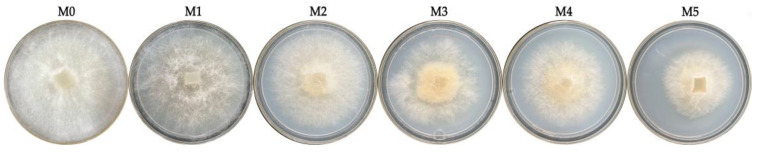
Mycelium phenotype of *V. volvacea* subcultured strains.

Five subculture strains, namely M1–M5 and one control strain, namely M0, were simultaneously inoculated on PDA medium and incubated at 30 °C for 72 h. The mycelium phenotype was recorded (Figure 1). The results showed that as the number of subcultures increased, the diameter of the mycelium gradually decreased, and the aerial hyphae gradually became sparse. The aerial hyphae of the M0 and M1 strains were dense and robust. The aerial hyphae of the M2 and M3 strains were dense, but the diameter of the mycelium was obviously reduced. The aerial hyphae density of the M4 strain decreased and the growth rate slowed down. The hyphae of M5 were severely twisted, and the colony shrank.

### 3.2. The Change in Fruiting Body Character of V. volvacea Subculture Strains

**Figure 2:**
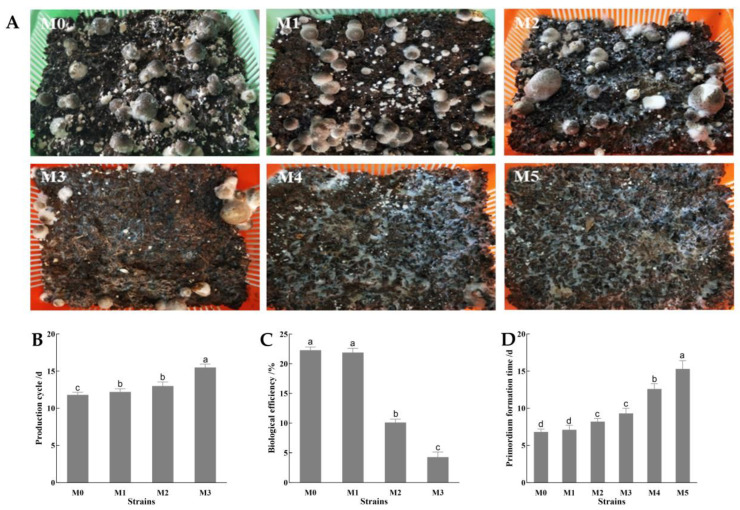
Production traits of *V. volvacea* subcultured strains. (**A**) *V. volvacea* cultivation; (**B**) production cycle; (**C**) biological efficiency of the fruiting bodies; (**D**) primordium formation time. The different lowercase letters denote significant differences among different stains (*p* < 0.05).

The basket planting test was performed for the M0-M5 strains in the laboratory, and the production characteristics of their fruiting bodies were recorded. The results showed that the shape of the fruiting bodies of the M0, M1, and M2 strains developed normally, while the number of fruiting bodies of the M2 and M3 strains decreased; these appeared as deformed mushrooms. The M4 and M5 strains could only form the primordium, and no fruiting bodies were observed (Figure 2A). Compared with M0, the production cycle and biological efficiency of the M1 strain were not significantly different (*p* > 0.05), the production cycle of strains M2 and M3 increased by 1.6 d and 3.7 d, respectively, and the biological efficiency of strains M2 and M3 significantly decreased by 54.71% and 80.72%, respectively (*p* < 0.05) (Figure 2B,C). The primordium formation time of the subcultured strains gradually extended as the number of subculture generations increased, and the primordium formation time of the M5 strain increased by 5.8 d compared with that of M0 (Figure 2D).

### 3.3. Sequencing Data Quality Analysis

The transcriptome sequencing of M1–M5 and M0 was performed using the RNA-seq technique. The results are shown in Table 2, after removing the joint and low-quality reads from the original sequence, more than 47 M clean reads were obtained from each sample. The Q30 base content of all samples was greater than 93%, and the proportion of GC base content was approximately 51.76 to 51.99%. The sequencing results were reliable and could meet the requirements of the subsequent data analysis.

### 3.4. Analysis of Differentially Expressed Genes in the V. volvacea Subcultured Strains

**Figure 3:**
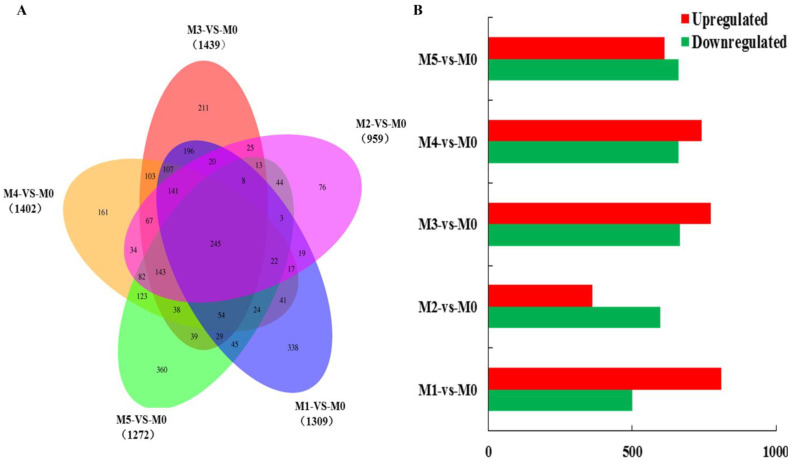
Differentially expressed genes in *V. volvacea* subcultured strains. (**A**) Venn diagram; (**B**) number of upregulated and downregulated genes.

With strain M0 being used as the control group, the number of differentially expressed genes in strains M1–M5 were counted, and the results are shown in Figure 3. Compared with M0, M3 had the largest number of differentially expressed genes, at 1439 (774 upregulated and 665 downregulated); this was followed by M4 at 1402 (741 upregulated and 661 downregulated genes). M2 had the lowest number of differentially expressed genes, at 959 (362 upregulated and 597 downregulated). The numbers of differentially expressed genes in M1 and M5 were 1309 and 1272, respectively. The total number of differentially expressed genes between M1–M5 and M0 was 245. The cultivation experiments showed that the strains subcultured after M3 all lost the ability to produce fruiting bodies, and that the highest number of differentially expressed genes was between M3 and M0. Therefore, repeated subculture for 12 months could significantly impact the degradation of *V. volvacea* strains.

### 3.5. GO Enrichment Analysis of Differentially Expressed Genes

**Figure 4:**
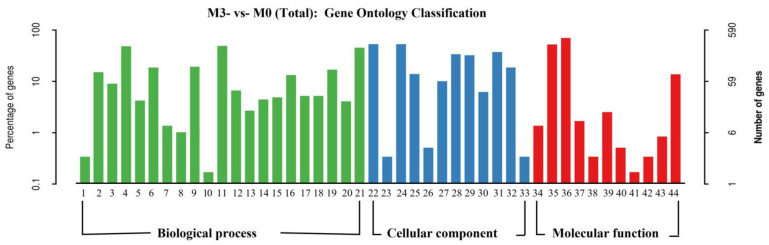
GO enriched analysis of differentially expressed genes. Note: 1—biological adhesion; 2—biological regulation; 3—cellular component organization or biogenesis; 4—cellular process; 5—developmental process; 6—establishment of localization; 7—growth; 8—immune system process; 9—localization; 10—locomotion; 11—metabolic process; 12—multi-organism process; 13—multicellular organismal process; 14—negative regulation of biological process; 15—positive regulation of biological process; 16—regulation of biological process; 17—reproduction; 18—reproductive process; 19—response to stimulus; 20—signaling; 21—single-organism process; 22—cell; 23—cell junction; 24—cell part; 25—extracellular region; 26—extracellular region part; 27—macromolecular complex; 28—membrane; 29—membrane part; 30—membrane-enclosed lumen; 31—organelle; 32—organelle part; 33—symplast; 34‚antioxidant activity; 35—binding; 36—catalytic activity; 37—enzyme regulator activity; 38—molecular transducer activity; 39—nucleic acid binding transcription factor activity; 40—nutrient reservoir activity; 41—protein binding transcription factor activity; 42—receptor activity; 43—structural molecule activity; 44—transporter activity.

GO enrichment analysis was performed on the genes that were differentially expressed between the M3 and M0 strains, and 44 functional groups were annotated; these were divided into three categories, namely biological processes, cell components, and molecular functions (Figure 4). Among them, the most differentially expressed genes were biological processes, with a total of 21 functional groups; these were mainly metabolic processes, cellular processes, biological regulation, single biological processes, biological process regulation, stress response, developmental processes, etc. There were 12 functional groups in cell components, and the most differentially expressed genes were organelle components, cells, cell membranes, and organelles. There were 11 functional groups in molecular function, and these were primarily catalytic activity, nucleic acid binding transcription factor activity, enzyme regulation activity, antioxidant activity, etc. The results of the GO enrichment analysis of differentially expressed genes between M1, M2, M4, and M5 and M0 were similar to those obtained for M3.

### 3.6. KEGG Enrichment Analysis of Differentially Expressed Genes

**Figure 5:**
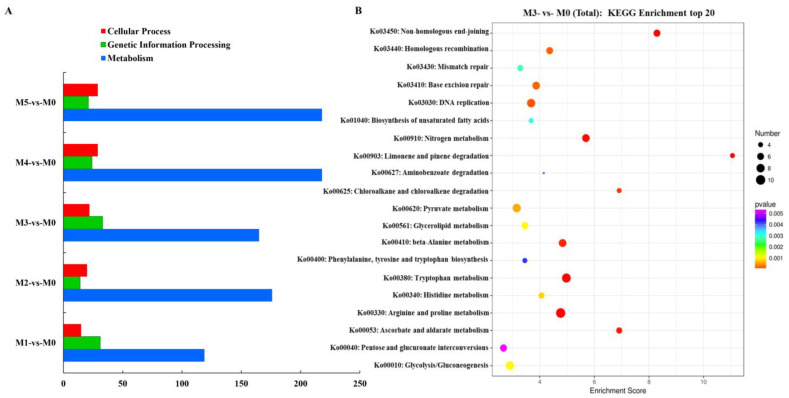
KEGG enrichment analysis of differentially expressed genes. (**A**) number of differentially expressed genes; (**B**) scatterplot of the top 20 differentially expressed genes between M3 and M0.

KEGG pathway enrichment analysis was performed on the differentially expressed genes. As shown in Figure 5A, the differentially expressed genes were significantly more enriched in metabolic pathways than in cellular processes and gene information processing processes. For M5 vs. M0 and M1 vs. M0, as the number of subcultures increased, the number of differentially expressed genes enriched in metabolic pathways and cellular processes gradually increased by 83.19% and 93.33%, respectively; in addition, the information processing gene decreased by 32.26%.

The genes that were differentially expressed between the M0 and M3 strains were mainly concentrated in the pathways of arginine and proline metabolism, tryptophan metabolism, β-alanine metabolism, nitrogen metabolism, ascorbic acid and aldarate acid metabolism, limonene and pinene degradation, non-homologous end junction, and DNA replication (Figure 5B).

### 3.7. Analysis of Differentially Expressed Genes Associated with Substrate Degradation

**Figure 6:**
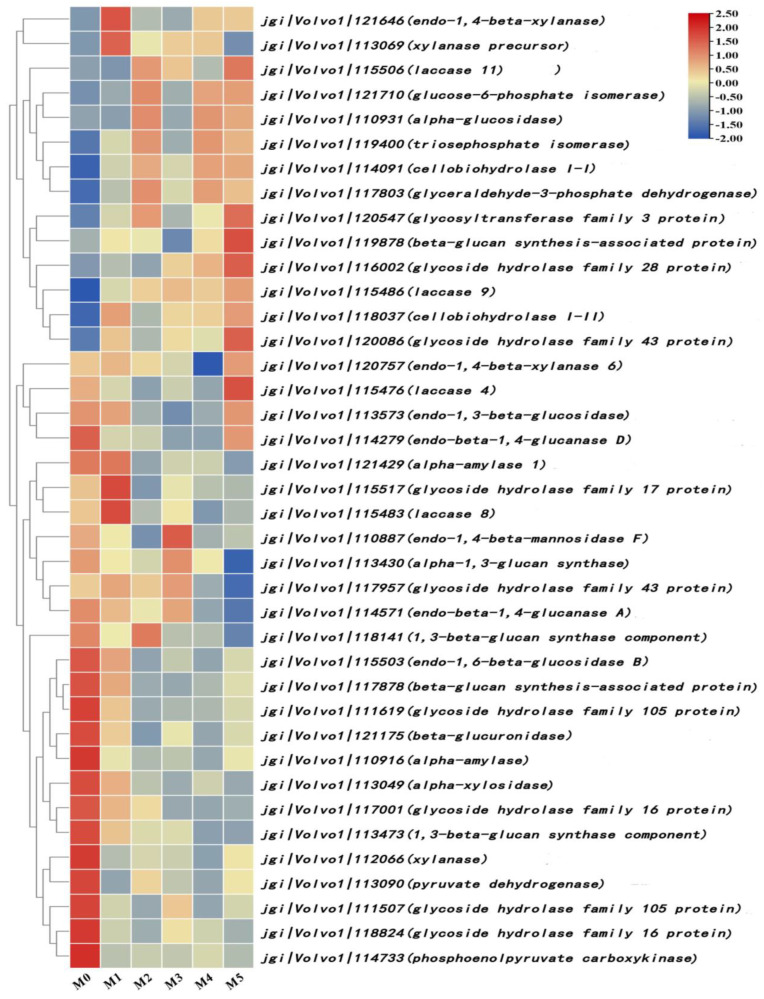
Differentially expressed genes related to substrate degradation.

After the further analysis and annotation of differentially expressed genes, 39 differentially expressed genes related to substrate degradation were screened out, as shown in Figure 6. The gene expression of cellobiohydrolase (jgi|Volvo1|114091, jgi|Volvo1|118037), α-glucosidase (jgi|Volvo1| 110931), and endonuclease-1,4-β-xylanase (jgi|Volvo1|121646) were upregulated. The gene expression of 1,3-β-glucan synthase (jgi|Volvo1|113473, jgi|Volvo1|118141), endomeric-1,6-β-glucosidase B (jgi|Volvo1|115503), endomeric-1,4-β-glucanase (jgi|Volvo1|114279), and endomeric-1,4-β-xylanase (jgi|Volvo1|120757) were upregulated. Among the laccase family genes, the gene expression of laccase-9 (jgi|Volvo1|115486) and laccase-11 (jgi|Volvo1|115506) was upregulated, whereas that of laccase-4 (jgi|Volvo1|115476) and laccase-8 (jgi|Volvo1|115483) was downregulated. The gene expression of α-amylase (jgi|Volvo1|110916, jgi|Volvo1|121429) was downregulated. The remaining 24 differentially expressed genes were all carbohydrate family-related genes indirectly involved in the degradation of the *V. volvacea* substrate; of these, nine were upregulated and fifteen were downregulated.

### 3.8. Analysis of Differentially Expressed Genes Related to Amino Acid Synthesis and Metabolism

**Figure 7:**
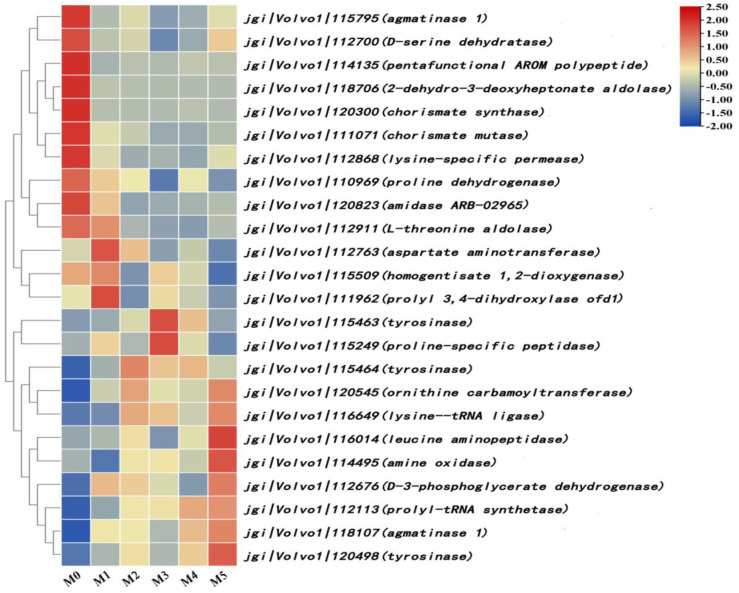
Differentially expressed genes related to amino acid synthesis and metabolism.

Twenty-four differentially expressed genes related to amino acid synthesis and metabolism were screened (Figure 7). Among them, genes involved in the biosynthesis of aromatic amino acids such as tyrosine and tryptophan (jgi|Volvo1|111071, jgi|Volvo1|114135, jgi|Volvo1|120300), serine (jgi|Volvo1|112700), arginine (jgi|Volvo1|115795), and proline (jgi|Volvo1|110969, jgi|Volvo1|111962, jgi|Volvo1|115249) were downregulated. The expression of genes related to serine (jgi|Volvo1|112676), proline (jgi|Volvo1|112113), and tyrosine (jgi|Volvo1|115463, jgi|Volvo1|115464, jgi|Volvo1|120498) metabolism was upregulated. The remaining 11 differentially expressed genes were also involved in amino acid synthesis and metabolism, including five upregulated genes and six downregulated genes. In addition, several differentially expressed genes involved in amino acid synthesis and metabolism were annotated as hypothetical proteins, so further studies on their functions are needed.

### 3.9. Analysis of Differentially Expressed Genes Related to Reactive Oxygen Metabolism

**Figure 8:**
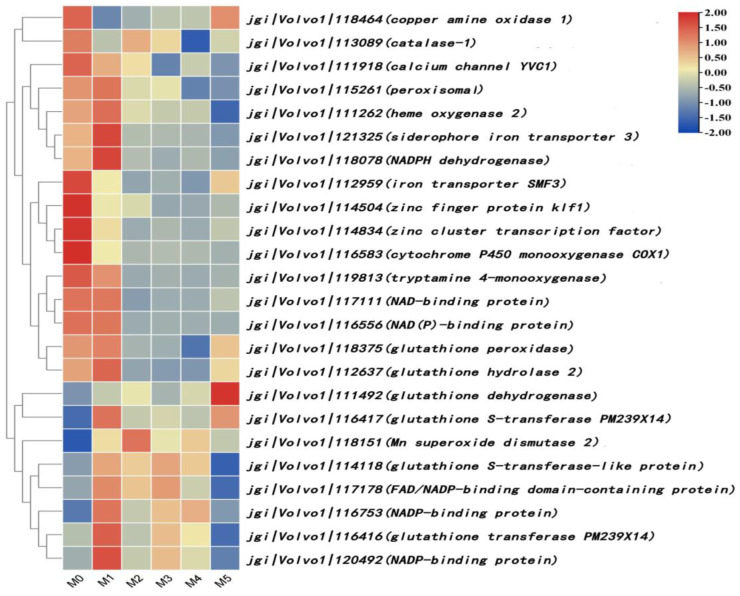
Differentially expressed genes related to the metabolism of reactive oxygen.

Twenty-four differentially expressed genes related to reactive oxygen metabolism were screened, as shown in Figure 8. Among them, the gene expression of glutathione peroxidase (jgi|Volvo1|118375), catalase-1 (jgi|Volvo1|113089), cytochrome P450 monooxygenase COX1 (jgi|Volvo1|116583), NADPH dehydrogenase (jgi|Volvo1|118078), glutathione hydrolase 2 (jgi|Volvo1|112637), and heme oxygenase 2 (jgi|Volvo1|111262) was downregulated. The gene expression of manganese superoxide dismutase 2 (jgi|Volvo1|118151), glutathione transferase PM239X14 (jgi|Volvo1|116416, jgi|Volvo1|116417), and glutathione dehydrogenase (jgi|Volvo1|111492) was upregulated.

Meanwhile, the gene expressions of the calcium channel (jgi|Volvo1|111918), iron transporter (jgi|Volvo1|121325, jgi|Volvo1|112959), and zinc finger protein (jgi|Volvo1|114504) were downregulated. The expression of the zinc finger protein gene in M3 was downregulated 13.09 times compared with M0. The remaining 10 differentially expressed genes, which are indirectly involved in reactive oxygen species metabolism in *V. volvacea*, include four upregulated genes and six downregulated genes.

### 3.10. RT-qPCR Validation of Differentially Expressed Genes

**Figure 9:**
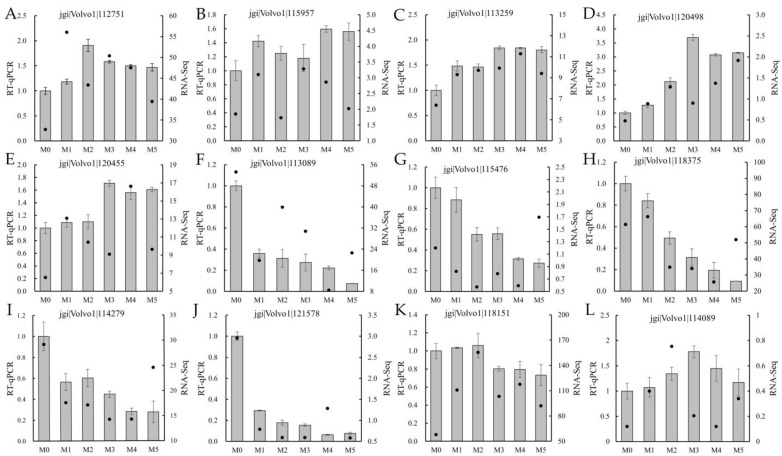
RT-qPCR confirmation of 12 differentially expressed genes. Note: the RNA-seq data are presented as log-transformed 2^−ΔΔCt^ values. The GPD gene was used as the internal reference gene to calculate the RNA-seq data. (**A**–**L**) were the relative expression and FPKM value of jgi|Volvo1|112751, jgi|Volvo1|115957, jgi|Volvo1|113259, jgi|Volvo1|120498, jgi|Volvo1|120455, jgi|Volvo1|113089, jgi|Volvo1|115476, jgi|Volvo1|118375, jgi|Volvo1|114279, jgi|Volvo1|121578, jgi|Volvo1|118151, jgi|Volvo1|114089, respectively.

In order to verify the reliability of transcriptomic sequencing data, 12 differentially expressed genes, involving three reactive oxygen metabolism genes, three substrate degradation genes, two gene mutation genes, two DNA methylation genes, one amino acid metabolism gene, and one toxin accumulation gene, were selected for RT-qPCR analysis, and the results are shown in Figure 9. The expression level five genes showed a trend of upregulation (Figure 9A–E), and the expression level of another five genes showed a trend of downregulation (Figure 9F–J); in the other two genes, the expression level was first upregulated and then downregulated (Figure 9K,L). The trends in the RT-qPCR analysis and transcriptome sequencing results of differentially expressed genes were generally consistent, indicating that the transcriptomic sequencing results were accurate.

## 4. Discussion

Edible mushroom strains are equivalent to the seeds of crops, and the quality of these strains directly affects the yield of edible mushrooms. The liquid paraffin preservation method is suitable for most edible mushrooms. This method enables mushrooms to be in storage for a long time, and enables the mycelium to grow normally after the residual paraffin has been removed [16]. However, this method requires a strict operating environment and advanced technology. In production, the simpler and more convenient method of periodic subculture preservation is widely used by producers, but repeated subcultures can lead to the degradation of strains. For example, it has been found that the virulence of *Metarhizium anisopliae* is reduced by repeated in vitro culturing [17]. The cellulase activity and carotene-producing ability of *Cordyceps militaris* decreased during the continuous subculture process. In degenerated strains of *Flammulina velutipes* after continuous subculturing, the growth of mycelium slowed down and the number of fruiting bodies reduced or became negligible [18]. In this study, as the subculture times increased, the diameter of the mycelium and the density of the aerial mycelium of *V. volvacea* subcultured strains decreased gradually. Cultivation experiments showed that the biological efficiency of the subcultured strains M2 and M3 decreased significantly, and that the production cycle was significantly prolonged. After 12 months of continuous subculturing, the M4 and M5 strains lost their ability to produce fruiting bodies.

*V. volvacea* is a typical straw decay mushroom that generally grows on agricultural waste such as saprophytic straw, wheat stalks, waste cotton, and cottonseed shells. The main components of these agricultural wastes are cellulose, hemicellulose, and lignin, etc., which provide sufficient carbon, nitrogen, and inorganic salts for the growth and development of *V. volvacea* [19,20]. When *V. volvacea* grows on the substrate, the mycelium attaches to the lignocellulose, enters the interior from the end, and begins to secrete cellulase, hemicellulase, lignin enzyme, etc. These enzymes work together to degrade macromolecules and obtain small molecules such as glucose to provide adequate nutrition and energy for mycelium growth and the development of the fruiting body [21,22]. The transcriptome sequencing performed in this study revealed that there are 39 differentially expressed genes related to substrate degradation in the *V. volvacea* subculture strains. The expression of major differentially expressed genes, such as 1,3-β-glucan synthase, endomeric-1,6-β-glucosidase B, endomeric-1,4-β-glucanase, endomeric-1,4-β-xylanase, laccase-4, laccase-8, etc., was downregulated; this could lead to the reduced degradation of lignocellulose and result in an insufficient supply of nutrients and energy, thus inhibiting the growth and formation of the fruiting bodies of *V. volvacea* subculture strains.

Amino acids are important nutrients for organisms. Amino acids could be used as organic nitrogen sources to promote the growth of edible mushrooms and as growth factors to regulate the metabolic activities of the organism [23]. Proline can reduce the levels of reactive oxygen species in edible mushrooms and prevent programmed cell death [24]. Arginine can regulate the growth and development of organisms via metabolites and various enzymes [25]. Serine is able to enter the one-carbon cycle and produce one-carbon units such as methyl, and is involved in purines and pyrimidine synthesis; this affects the conversion of various amino acids and nutrients [26]. Tyrosine and tryptophan have certain antioxidant effects due to their special -OH and -NH structural units [27]. This study showed that the expression levels of differentially expressed genes involved in the synthesis of tyrosine, tryptophan, serine, arginine, and proline all showed a trend of downregulation (Figure 7). This suggests that the continuous subculture of *V. volvacea* might lead to the inhibition of amino acid synthesis within the mycelium, thus further affecting the growth of mycelia and the formation of fruiting bodies.

Reactive oxygen species are produced by aerobic organisms during the metabolic process, and are crucial for the growth of edible mushrooms; they also affect the formation of the primordia [28] and the development of the fruiting body [29]. In healthy organisms, the production and removal of reactive oxygen species are balanced. However, when organisms are stimulated by external factors, undergo pathological changes, or age, this balance can be destroyed, causing a rapid increase in reactive oxygen species. If these excess reactive oxygen species are not removed in time, serious damage can be caused to DNA, proteins, lipids, and other substances in the organism [30]. To avoid this damage, organisms have developed a system involving antioxidant enzymes that clears reactive oxygen species [31]. In this study, the expression of antioxidant enzyme genes such as glutathione peroxidase and catalase was downregulated as the time required for *V. volvacea* subculture increased. Some mineral elements are cogroups, cofactors, or activators of antioxidant enzymes, which increase enzyme activity and promote enzyme action [32]. In this study, the differentially expressed genes related to the calcium channel, iron transporter, and zinc finger protein were downregulated. This may affect the activity of antioxidant enzymes, resulting in the excessive accumulation of reactive oxygen species in subculture strains, thus inhibiting the growth and accelerating the degradation of the *V. volvacea* strain.

At present, several studies have been conducted on the degradation mechanisms of the strain. The glycosylation of mitochondrial DNA and oxidative stress could lead to the degradation of *M. anisopliae* [33]. The viral infection of the mycoplasma could lead to the strain degradation of *Pleurotus ostreatus* [34]. Genetic mutation [35,36], methylation modification [37,38], and the accumulation of toxins [39] can also lead to the degradation of edible mushroom stains. In this study, the expression levels of differentially expressed genes increased as the number of *V. volvacea* subcultures increased; these included ATP deconjugulase IRC5, E3 ubiquitin–protein ligase, and DNA methyltransferase. The expression levels of the ethanol oxidase gene showed a decreasing trend (Figure 9). This may also be one reason for the degradation of the *V. volvacea* strain.

In this study, we conducted a transcriptomic analysis of the degradation of *V. volvacea* subculture strains. After the continuous subculture of *V. volvacea* mycelium, the expression levels of some differentially expressed genes related to substrate degradation, such as amino acid synthesis and metabolism, as well as reactive oxygen clearance, showed a decreasing trend. This could lead to the excessive accumulation of reactive oxygen *V. volvacea*; the inhibition of nutrient absorption and energy acquisition, which can result in the occurrence of degradation phenomena such as the inhibition of mycelial growth; a decrease in the production of substrates; and a prolonged production cycle. These findings could form the theoretical basis of further studies on the degeneration mechanism of *V. volvacea*, and also provide a basis for molecular function studies of the genes related to strain degradation.

## Figures and Tables

**Table 1:** Primers used for RT-qPCR.

Gene Symbol	Description	Primer Sequence (5′→3′)
jgi|Volvo1|112751	E3 ubiquitin–protein ligase	F: AGTTCACGCAATGCGAATAR: TCATCTGGATCGTGTGTAATGG
jgi|Volvo1|113089	catalase (CAT)	F: AGGCTCTGACATTCATCATTGR: AAAGCAGCTTCACCCATAC
jgi|Volvo1|113259	DNA-methyltransferase 1A	F: TGTGCTCAAGAACCAACCTAR: CCCTGGGCAATACGGGATA
jgi|Volvo1|114089	cellobiohydrolase I-I	F: ACAAACGGAATCACAACTAGCR: CGCGTGACACAGTATCAG
jgi|Volvo1|114279	endo -beta-1,4-glucanase D	F: TTATTCCCGATATTCCTGCTGGR: TTCTGAGAGTACAGTGCGT
jgi|Volvo1|115957	DNA-methyltransferase 1B	F: CGACGATGGATTTACAGCATTAR: TTGCCACTCGTACAAGTCAG
jgi|Volvo1|118151	Mn superoxide dismutase (SOD)	F: CACAAAGACCGCTGCTATCR: TAGTAACGACCTCTAGCTTGC
jgi|Volvo1|118375	glutathione peroxidase (GPX)	F: TCGGAGGTGAATGGGAACR: TTGATCCTCGTCAGACCCATA
jgi|Volvo1|120455	ATP-dependent helicase IRC5	F: CCAACGCGAAGCCTATAACR: GCTCCTCATCTTTCTTAGCTG
jgi|Volvo1|121578	aryl alcohol oxidase	F: GAATCCGTTCTCCCTCAAGR: GCGCAGTGGTAGTGTAAT
jgi|Volvo1|115476	laccase-4	F: ATGCGGTTCTGGTCAATGR: TTGTGTGACAGAGAGTTCGT
jgi|Volvo1|120498	tyrosinase	F: GGGCGAAACTGAACTGGR: CTGGGAGCGTCATAGGA
jgi|Volvo1|117803	glyceraldehyde-3-phosphate dehydrogenase (GPD)	F: GGCTTGATGACCACCGTACATR: GCACCAGTGGAAGATGGAATAATG

**Table 2:** Quality statistics of the sequencing data.

Strains	M0	M1	M2	M3	M4	M5
Raw reads M	49.55	49.37	49.38	49.48	49.42	49.55
Clean reads M	48.21	48.12	47.92	48.14	47.97	48.09
Valid bases %	93.45	0.93	93.53	93.61	93.36	92.45
Q30 content %	93.50	93.75	93.95	94.30	94.28	94.06
GC content %	51.99	51.89	51.89	51.83	51.97	51.76
Total mapped reads %	97.45	97.00	97.23	96.80	97.61	97.39

Note: the Q30 content is the percentage of bases with a mass value greater than 30 in the total base. The GC content is the sum of the numbers of G and C as a percentage of the total number of bases; the total mapped reads is the number of sequences mapped to the reference genome.

## Data Availability

The original contributions presented in the study are included in the article and Supplementary Materials; further inquiries can be directed to the corresponding author.

## Supplementary Materials

The following supporting information can be downloaded at https://www.mdpi.com/article/10.3390/jof11010007/s1, Supplementary Figure S1, the sketch map of how to obtain M1–M5, the five subculture strains.

## References

[1] Lyu X., Jiang S., Wang L., Chou T., Wang Q., Meng L., Mukhtar I., Xie B., Wang W. (2021). The Fvclp1 gene regulates mycelial growth and fruiting body development in edible mushroom *Flammulina velutipes*. Arch. Microbiol..

[2] Guillamon E., Garcia-Lafuente A., Lozano M., D’Arrigo M., Rostagno M.A., Villares A., Alfredo Martinez J. (2010). Edible mushrooms: Role in the prevention of cardiovascular diseases. Fitoterapia.

[3] Chen S., Ma D., Ge W., Buswell J.A. (2003). Induction of laccase activity in the edible straw mushroom, *Volvariella volvacea*. FEMS Microbiol. Lett..

[4] Li N., Chen F., Cui F., Sun W., Zhang J., Qian L., Yang Y., Wu D., Dong Y., Jiang J. (2017). Improved postharvest quality and respiratory activity of straw mushroom (*Volvariella volvacea*) with ultrasound treatment and controlled relative humidity. Sci. Hortic..

[5] Hou L., Li Y., Chen M., Li Z. (2017). Improved fruiting of the straw mushroom (*Volvariella volvacea*) on cotton waste supplemented with sodium acetate. Appl. Microbiol Biot..

[6] Agreda T., Cisneros O., Agueda B., Marina Fernandez-Toiran L. (2014). Age class influence on the yield of edible fungi in a managed mediterranean forest. Mycorrhiza.

[7] Homolka L. (2014). Preservation of live cultures of basidiomycetes—Recent methods. Fungal Biol..

[8] Bao D.P., Gong M., Zheng H.J., Chen M.J., Zhang L., Wang H., Jiang J.P., Wu L., Zhu Y.Q., Zhu G. (2013). Sequencing and comparative analysis of the straw mushroom (*Volvariella volvacea*) genome. PLoS ONE.

[9] Liu M., Yu T., Singh P.K., Liu Q., Liu H., Zhu Q., Xiao Z., Xu J., Peng Y., Fu S. (2020). A comparative transcriptome analysis of *Volvariella volvacea* identified the candidate genes involved in fast growth at the mycelial growth stage. Genes.

[10] Chen X., Zhang Z., Liu X., Cui B., Miao W., Cheng W., Zhao F. (2019). Characteristics analysis reveals the progress of *Volvariella volvacea* mycelium subculture degeneration. Front. Microbiol..

[11] Kim D., Langmead B., Salzberg S.L. (2015). HISAT: A fast spliced aligner with low memory requirements. Nat. Methods.

[12] Kanehisa M., Araki M., Goto S., Hattori M., Hirakawa M., Itoh M., Katayama T., Kawashima S., Okuda S., Tokimatsu T. (2008). KEGG for linking genomes to life and the environment. Nucleic Acids Res..

[13] Young M.D., Wakefield M.J., Smyth G.K., Oshlack A. (2010). Gene ontology analysis for RNA-seq: Accounting for selection bias. Genome Biol..

[14] Wang W.P., Tan Q.F., Cheng Z.H., Sun W.H., Yuan J.M., Zhao F.Y. (2022). Selection of optimal RT-qPCR reference genes for examining different strains of *Volvariella volvacea*. Mycosystema.

[15] Livak K.J., Schmittgen T.D. (2001). Analysis of relative gene expression data using real-time quantitative PCR and the 2(-Delta Delta C(T)) Method. Methods.

[16] Little G.N., Gordon M.A. (1967). Survival of fungus cultures maintained under mineral oil for twelve years. Mycologia.

[17] Shah F.A., Allen N., Wright C.J., Butt T.M. (2007). Repeated in vitro subculturing alters spore surface properties and virulence of *Metarhizium anisopliae*. FEMS Microbiol. Lett..

[18] Kim S.Y., Kim K.-H., Im C.H., Ali A., Lee C.Y., Kong W.-S., Ryu J.-S. (2014). Identification of degenerate nuclei and development of a SCAR marker for *Flammulina velutipes*. PLoS ONE.

[19] Baldrian P., Valaskova V. (2008). Degradation of cellulose by basidiomycetous fungi. FEMS Microbiol. Rev..

[20] Wang Z., Lehr N., Trail F., Townsend J.P. (2012). Differential impact of nutrition on developmental and metabolic gene expression during fruiting body development in *Neurospora crassa*. Fungal Genet. Biol..

[21] Lynd L.R., Weimer P.J., van Zyl W.H., Pretorius I.S. (2002). Microbial cellulose utilization: Fundamentals and biotechnology. Microbiol. Mol. Biol. Rev. MMBR.

[22] Neumann J., Matzner E. (2013). Biomass of extramatrical ectomycorrhizal mycelium and fine roots in a young Norway spruce stand—A study using ingrowth bags with different substrates. Plant Soil..

[23] Yang G., Wei Q., Huang H., Xia J. (2020). Amino Acid Transporters in Plant Cells: A Brief Review. Plants.

[24] Szabados L., Savoure A. (2010). Proline: A multifunctional amino acid. Trends Plant Sci..

[25] Li B., Ding Y., Tang X., Wang G., Wu S., Li X., Huang X., Qu T., Chen J., Tang X. (2019). Effect of L-arginine on maintaining storage quality of the white button mushroom (*Agaricus bisporus*). Food Bioprocess Technol..

[26] Ros R., Munoz-Bertomeu J., Krueger S. (2014). Serine in plants: Biosynthesis, metabolism, and functions. Trends Plant Sci..

[27] Waheed A., Zhuo L., Wang M., Hailiang X., Tong Z., Wang C., Aili A. (2024). Integrative mechanisms of plant salt tolerance: Biological pathways, phytohormonal regulation, and technological innovations. Plant Stress.

[28] Zhang J., Chen H., Chen M., Wang H., Wang Q., Song X., Hao H., Feng Z. (2017). Kojic acid-mediated damage responses induce mycelial regeneration in the basidiomycete *Hypsizygus marmoreus*. PLoS ONE.

[29] Zhu J., Wang W., Sun W., Lei Y., Tan Q., Zhao G., Yun J., Zhao F. (2024). Overexpression of cat2 restores antioxidant properties and production traits in degenerated strains of *Volvariella volvacea*. Free Radic. Biol. Med..

[30] Hasanuzzaman M., Bhuyan M.H.M.B., Zulfiqar F., Raza A., Mohsin S.M., Al Mahmud J., Fujita M., Fotopoulos V. (2020). Reactive oxygen species and antioxidant defense in plants under abiotic stress: Revisiting the crucial role of a universal defense regulator. Antioxidants.

[31] Zhang J., Hao H., Chen M., Wang H., Feng Z., Chen H. (2017). Hydrogen-rich water alleviates the toxicities of different stresses to mycelial growth in *Hypsizygus marmoreus*. Amb. Express.

[32] Santos T., Connolly C., Murphy R. (2015). Trace element inhibition of phytase activity. Biol. Trace Elem. Res..

[33] Li L., Pischetsrieder M., St Leger R.J., Wang C. (2008). Associated links among mtDNA glycation, oxidative stress and colony sectorization in *Metarhizium anisopliae*. Fungal Genet. Biol..

[34] Qiu L., Li Y., Liu Y., Gao Y., Qi Y., Shen J. (2010). Particle and naked RNA mycoviruses in industrially cultivated mushroom *Pleurotus ostreatus* in China. Fungal Biol..

[35] Yin J., Xin X.D., Weng Y.J., Gui Z.Z. (2017). Transcriptome-wide analysis reveals the progress of *Cordyceps militaris* subculture degeneration. PLoS ONE.

[36] Tuteja N., Tuteja R. (2004). Prokaryotic and eukaryotic DNA helicases. Essential molecular motor proteins for cellular machinery. Eur. J. Biochem..

[37] Xin X., Yin J., Zhang B., Li Z., Zhao S., Gui Z. (2019). Genome-wide analysis of DNA methylation in subcultured *Cordyceps militaris*. Arch. Microbiol..

[38] Zhang H., Lang Z., Zhu J.-K. (2018). Dynamics and function of DNA methylation in plants. Nat. Rev. Mo. Cell Biol..

[39] Dinh T.N., Nagahisa K., Hirasawa T., Furusawa C., Shimizu H. (2008). Adaptation of Saccharomyces cerevisiae cells to high ethanol concentration and changes in fatty acid composition of membrane and cell size. PLoS ONE.

