# Supplementary figures and images for: Molecular Mechanism During Mycelium Subculture Degeneration of Volvariella volvacea
Source: J Fungi (Basel). 2024 Dec 25;11(1):7. doi: 10.3390/jof11010007 (PMC11766388; doi:10.3390/jof11010007)

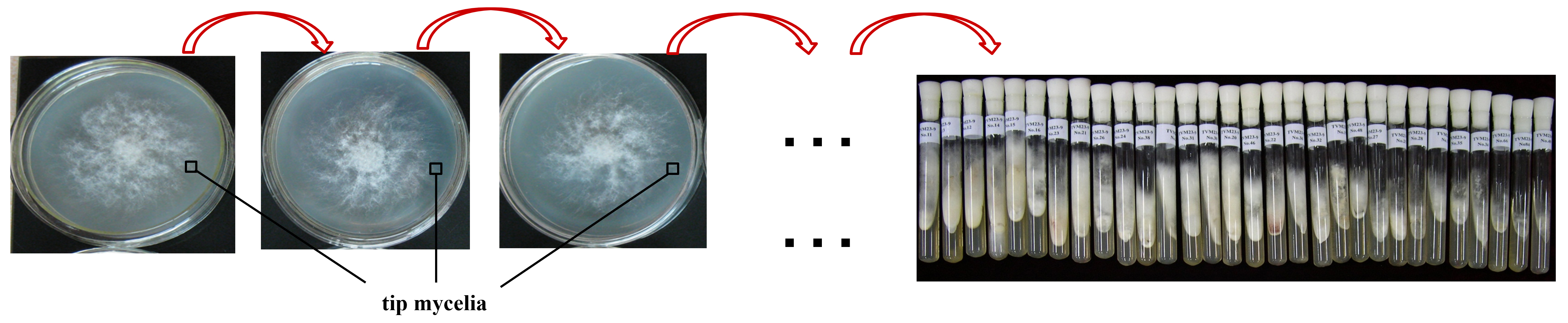

Supplement: Supplementary file 1 [file jof-11-00007-s001.zip › Supplementary Figure S1.jpg]
